# Eye Movement Desensitization and Reprocessing: Efficacy in Improving Clinical, Neuropsychological, and Quality of Life in Women Victims of Violence

**DOI:** 10.1089/whr.2023.0110

**Published:** 2024-12-06

**Authors:** Alexandra Yakeline Meneses Meneses, Sol Fernández-Gonzalo, Mercè Jodar Vicente

**Affiliations:** ^1^ School of Psychology and Education, Universidad de Las Américas, Quito, Ecuador.; ^2^ Department of Clinical and Health Psychology, Universitat Autònoma de Barcelona, Barcelona, Spain.; ^3^ Instituto de Investigación e Innovación Parc Tauli-I3PT, Sabadell, Spain.; ^4^ CIBERSAM, Instituto de Salud Carlos III, Madrid, Spain.; ^5^ Neurology Service, Hospital Universitario Parc Tauli, Sabadell, Spain.

**Keywords:** battered women, EMDR therapy, gender-based violence, NET therapy, post-traumatic stress, quality of life

## Abstract

**Background:**

The number of female victims of violence has significantly increased in recent years, resulting in physical, mental, and social damage.

**Objective:**

To determine the effectiveness of the eye movement desensitization and reprocessing (EMDR) psychotherapeutic model compared with narrative exposure therapy (NET) as treatments for clinical improvement, neuropsychological outcomes, and quality of life in women who have experienced violence.

**Methods:**

A randomized experimental study was conducted, involving 120 women exposed to physical, psychological, and sexual violence, who were assigned to either an EMDR or NET group. An extensive battery of clinical, neuropsychological, and quality of life tests was administered both before and after a 10-session therapeutic intervention.

**Results:**

Compared with the group of women treated with NET, the group of women who received EMDR therapy, exposed to physical, psychological, and sexual violence, achieved a greater decrease in anxiety (*p* = 0.001), depression (*p* = 0.001), and post-traumatic symptoms (*p* = 0.002). Additionally, there was an increase in the quality of life index (*p* = 0.001), performance in working memory (*p* = 0.000), and executive functioning tests (*p* = 0.000), compared with NET.

**Conclusions:**

EMDR proved to be more effective compared with NET in reducing post-traumatic clinical symptoms, increasing the level of quality of life, and enhancing cognitive performance in women affected by gender-based violence. Additionally, it demonstrated independence in therapeutic response across most estimated sociodemographic factors, making it a therapy with broader therapeutic reach in the community of Ecuadorian women.

## Introduction

Violence against women in all its forms is considered a violation of human rights, a public health issue, and an emerging area of social concern. Therefore, it represents one of the most significant challenges facing today’s society.^1^ Currently, one in three women (30%) reports having experienced physical or sexual violence from their romantic partners, ex-partners, or third parties, providing evidence of the need to recognize the negative impact that this abuse has on the overall health of affected women.^2^

Several studies have shown the importance of addressing this problem.^3^ Recently, research has emphasized the study of the negative effects of violence against women, including physical, mental, and sexual violence.^4–6^ Moreover, there is a focus on understanding the influence of social roles and culture on the perception of gender violence.^7^ Other studies have concentrated on the psychomotor alterations that result from brain injuries.^8^

In Latin American countries, there are reports of higher incidences of violence against women. In Ecuador, 6 out of 10 women have reported gender-based violence, mostly originating from their male romantic partners. These higher incidences are associated with the environment in which cultural, social, religious, and traditional patterns develop.^9^ Hence, violence against women represents a serious health problem that leads to psychopathological and cognitive alterations, and even death.^10^ There are several aspects that exert influence to varying degrees of vulnerability, often mediated by sociodemographic and socioeconomic factors.^11^

A latent association has been identified between clinical symptoms and cognitive functioning in this group of women.^12^ In this sense, women who report anxiety, depression, post-traumatic stress, or exposure to sexual violence tend to exhibit low performance in neuropsychological evaluations, especially in attentional, visuospatial, and executive tasks.^13,14^ Additionally, there is evidence that sociodemographic factors are related to the adverse effects of violence on the physical and mental health of female victims, as well as on their quality of life.^15,16^

Consequently, given the reality surrounding this problem, over the last 5 years, intensive treatments have emerged for women who experience trauma associated with exposure to physical, psychological, and sexual violence. The goal is to address and overcome the physical and mental effects caused.^17,18^ Many studies have shown therapeutic treatments with benefits at the cognitive, emotional, and social levels in female victims; however, they have not yet demonstrated comprehensive effectiveness among patients.^19,20^

In response to the issue of violence against women and its effects, within the context of the Ecuadorian public health system, policies, laws, and regulations have been implemented to address cases of female patients seeking healthcare services and reporting having been victims of physical, psychological, and sexual violence. This is considered a priority group according to the Constitution of the Republic of Ecuador.^21^ Within the models of mental health care specific to the Ecuadorian context, protocols for psychological first aid and comprehensive therapeutic follow-up for victims are outlined. The therapy models predominantly used by the healthcare system are derived from the systems approach, from which the techniques employed in narrative exposure therapy (NET) originate.

NET is accredited as a successful and culturally universal intervention for the treatment of survivors of multiple and severe traumatic events.^22^ This intervention, based on connecting traumatic memories, facilitates the reevaluation of events and cognitive reorganization by organizing a chronological timeline of the painful events experienced by the patient. This allows them to narrate traumatic experiences, while finding new meanings, embracing both positive and negative aspects, and assessing their level of resilience to reshape their life story.^23^ Therefore, NET has been more frequently utilized in community settings and with individuals who have experienced trauma due to political, cultural, or social forces. The effectiveness of NET has been reported in reducing post-traumatic stress, depression, and improving the quality of life in such cases.^24,25^

However, while NET has shown positive results in reducing depression and post-traumatic symptoms, it is not known to what extent this therapy affects other dimensions of the health of victims.^26^ Also, to the best of our knowledge, its effectiveness in reducing the negative symptoms experienced by victims of violence has not been validated. Therefore, it is noteworthy that the World Health Organization^27^ published a new protocol and clinical guidelines for healthcare workers addressing the treatment of the mental health repercussions of trauma and the loss of loved ones. Among the cited approaches are “cognitive-behavioral therapy or a new technique known as Eye Movement Desensitization and Reprocessing (EMDR).” Additionally, the National Institute for Care Excellence ^28^ recommended the use of EMDR for the treatment of post-traumatic stress disorder in adults.

Recent studies have reported the benefits of EMDR on the physical and mental health of women victims of violence.^29–31^ Thus, international treatment guidelines recommend this therapy as a first-line treatment for post-traumatic stress disorder, considering it as effective as trauma-focused cognitive-behavioral therapy in treating depression and post-traumatic stress disorder.^32^

Despite these findings, nothing has been done to implement these new therapies in the Ecuadorian public health system, indicating a lack of interest in providing quality mental health care focused on reducing the adverse symptoms accompanying victims. Many of them continue to experience post-traumatic stress throughout their lives. Based on this analysis, the idea arises to investigate with a group of Ecuadorian women seeking care in the Ecuadorian public health service, applying conventional therapeutic treatment based on NET and EMDR therapy. With this, we aim to answer the following question: How effective is EMDR compared with NET in reducing clinical symptoms, improving cognitive performance, and enhancing the quality of life in women who have experienced violence and received therapeutic treatment?

Against this background, the objective of this study was to assess the effectiveness of EMDR compared with NET as treatments for clinical improvement, neuropsychological outcomes, and quality of life in women victims of violence.

## Methodology

### Design

This study was framed within an open randomized experimental design; the intervention is based on the methodology for health studies with two comparative groups with pre–post repeated measures, through which we sought to determine the effect of an intervention.^33^

### Participants

**FIG. 1.**
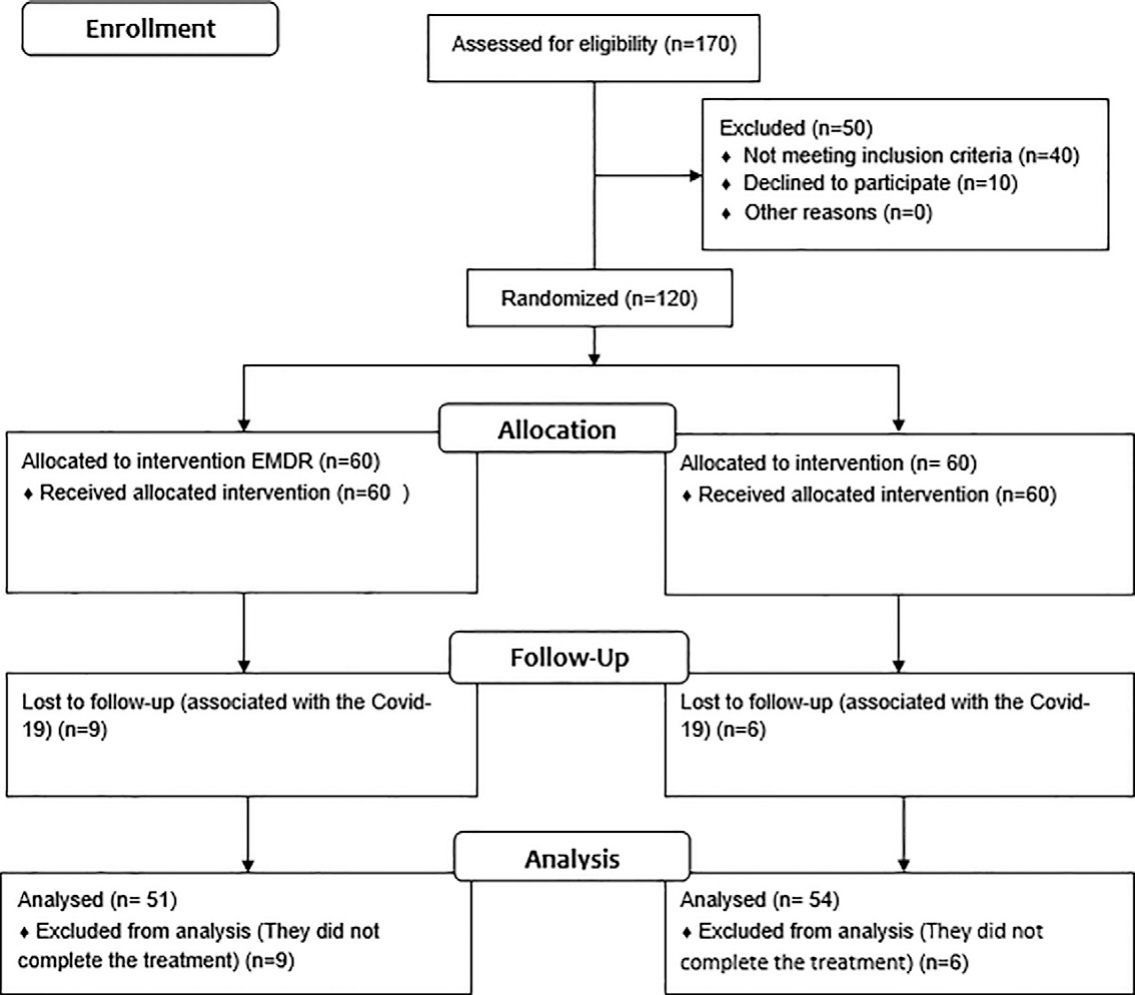
CONSORT flow diagram.

As shown in Figure 1, 170 women were assessed for participation, all of whom reported a history of physical, psychological, and sexual gender-based violence when seeking psychology services. From this identified group, 50 women were excluded because of inability to travel, current participation in other treatment plans, or declining because of lack of time to participate.

The result of this selection process yielded a total of 120 women exposed to gender-based violence, with an average age of 34.3 years and a low level of education. The average duration of systematic exposure to violence was 6.4 years, with the majority experiencing physical violence. About 41.89% of the participants lived with a male partner. These women were identified and invited to participate in the study through professionals from the public health services of the Tabacundo Health Center in Ecuador. They were then randomly divided into two treatment groups. Inclusion criteria were: (1) exposure to gender-based violence, whether physical, psychological, and/or sexual; (2) age between 18 and 50; and (3) signed informed consent. Exclusion criteria were: (1) intelligence quotient (IQ) below 70; (2) history of brain injury; (3) history of neurological disease; (4) schizophrenia or other primary psychotic disorders as described in the International Classification of Diseases–eleventh edition (ICD-11), (5) substance use disorders or addictive behaviors, including all substances described in the ICD-11; and (6) visual and auditory disabilities.

### Procedure

This study was approved by the health committee of District 17D10, Cayambe—Pedro Moncayo, and by the Committee of Ethics and Research with Human Beings of the Universidad Tecnológica Equinoccial (UTE). All selected women were informed about the characteristics and procedures of the study and the voluntary nature of their participation, and they signed an informed consent form. Women who agreed to participate in the study were administered the Spanish version of the National Adult Reading Test (NART)^34,35^ to rule out intellectual disability. Those who met the inclusion criteria underwent the pretest, which consisted of a complete battery to evaluate the clinical profile, cognitive functions, and quality of life of the participants.

Subsequently, the women were assigned to the corresponding therapy groups: Group A—60 women who received EMDR therapy; and Group B—60 women who received NET. Dropouts that occurred during the treatment phase were considered in the analysis (Fig. 1). Following the protocols for the treatment plan designed for each group and the recommendations of the ethics committee, two specialized therapists were assigned to a random list of participants based on the therapist’s specialty, either EMDR or NET. Ten personalized therapeutic sessions were scheduled, each lasting 60 minutes.

### Therapeutic interventions

For the group that received EMDR therapy, the standard protocol was applied^36,37^ based on the adaptive information processing states,^38^ which consists of eight treatment phases. The techniques used were those indicated in the same standard EMDR protocol. The following desensitization and reprocessing techniques were implemented: float back, float forward, affective scan, dual attention,^39^ bilateral auditory stimulation,^40^ and bilateral visual stimulation.^41^ Additionally, stress management techniques, exercises based on mental games for cognitive stimulation, and cognitive interweaving for the management of complex targets were employed. A description of the tasks used for each phase of the protocol is provided in Table 1.

**Table 1.** Description of the Tasks Used in the Eye Movement Desensitization and Reprocessing Treatment Phases

Phases	Tasks
*Phase 1* *History*	• Develop the narrative or structured history. • Conduct an objective evaluation of symptoms. • Identify targets for reprocessing: past aetiological events for current symptoms, current triggers, and future objectives.
*Phase 2* *Preparation*	Guide the participant through trauma-based psychotherapy with EMDR. Provide metaphors for conscious observation in the reprocessing process. Verify the usefulness of self-control methods through the diary of the participant.
*Phase 3* *Evaluation*	Assess images, negative beliefs that currently govern, desired positive beliefs, emotions, and physical sensations. Record initial SUD and VoC scores.
*Phase 4* *Desensitization*	Perform separate series of bilateral stimulation, and evaluate changes through brief reports from the participant. Return to the target or incident periodically to evaluate changes and identify residual issues. Make use of additional interventions if a blocked reprocessing is identified.
*Phase 5* *Installation*	Conduct separate series of bilateral stimulations, while the patient maintains consciousness of the target. Continue until the moment when the participant reaches a VoC of 7 or 6.
*Phase 6* *Examination of the body*	Conduct separate series of bilateral stimulations when the participant focuses on reprocessing any residual physical sensations and begins to have positive or neutral sensations.
*Phase 7* *Closing*	If necessary, use self-control techniques that guarantee stability and orientation. Report the effects of the treatment to the participant. Request a personal journal of observations between sessions.
*Phase 8* *Reassessment*	Adjust the treatment plan Check the target to ensure effects and assess stability.

Based on the EMDR Standard Protocol Guide.^36,37^

EMDR, eye movement desensitization and reprocessing.

Similarly, for the group undergoing NET, the treatment model outlined was applied.^24^ Table 2 illustrates the structured intervention with four practical elements: (1) diagnostic interview, (2) psychoeducation, (3) lifeline description (symbolic representation), (4) commencement of NET therapy sessions, and (5) rituals for the final session.^42^

**Table 2.** Description of Essential Components for Narrative Exposure Therapy

Element	Activity
1	Diagnostic interview: Psychoeducation
2	Description of lifeline (symbolic representation) (a) Actively reconstructing the chronological order of autobiographical/episodic memory. (b) Engaging in imaginary exposure to the traumatic events (identified “hot spots”).
3	Initiation of NET sessions (a) Creating meaningful connections and blending physiological, sensory, cognitive, and emotional responses within the framework of one’s time, space, and life context. (b) Reassessing cognitive aspects of behavior and patterns (*e.g.,* cognitive distortions, automatic thoughts, beliefs, responses), and reinterpreting the meaning/content by reprocessing negative, fearful, and traumatic events until resolution and closure.
4	Final session rituals (a) Reflecting on positive life experiences to activate resources and adapt fundamental assumptions. (b) Restoring the survivor’s dignity by addressing the need for recognition, emphasizing the explicit human rights orientation in providing testimony.

Based on the emerging evidence for NET.^23,24^

NET, narrative exposure therapy.

### Instruments

#### Survey on gender violence

Based on the Technical Standard for Comprehensive Attention to Gender Violence,^43^ it allows the collection of data associated with sociodemographic variables such as age, gender, marital status, and economic status. Additionally, it includes information on the type of reported violence and the duration of exposure to violence.

#### National adult reading test

This is a test for reading unstressed words, which correlates with the QI index of the WAIS intelligence test and allows estimating the QI.^34^ This test was administered with the aim of ruling out potential intellectual limitations in the participants, as part of the exclusion criteria for the sample, using the Spanish-adapted version of the Word Accentuation Test.^35^ The premorbid IQ is scored according to the direct form of 0/30, where a score of 10 on NART equals 31 on WAIS-IV, and a score of 30 equals 136 on WAIS-IV. The Cronbach’s alpha coefficient of validity is (0.84).

#### Hospital anxiety and depression scale

It allows the assessment of the levels of anxiety and depression that the subject presents.^44^ It consists of seven items related to anxiety symptoms and seven items related to depressive symptoms, with each item valued on a scale of 0–3 points. The cutoff point is seven. Subscale scores for anxiety: 0–7: no anxiety; 8–10: minimal anxiety; and 11–21: clinical anxiety. Subscale scores for depression: 0–7: no depression; 8–10: minimal depression; and 11–21: clinical depression. The Cronbach’s alpha coefficient of validity is (0.76).

#### Post-traumatic stress disorder checklist for DSM-5

It is an instrument updated to the DSM-V to evaluate post-traumatic symptoms.^45^ Respondents indicate how much each PTSD symptom has disturbed them in the past week (as opposed to the past month), using a five-point scale ranging from 0 = not at all, 1 = a little, 2 = moderately, 3 = quite a bit, and 4 = extremely. The cutoff point is 31. Note 1: The patient must previously meet Criterion A. Note 2: A total score of 31–33 points is optimal to determine probable PTSD. Note 3: To determine whether or not it meets the Criterion for a Provisional Diagnosis of PTSD, we will only consider symptoms with scores of 2, 3, or 4. The Cronbach’s alpha coefficient of validity is (0.90–0.97).

#### GENCAT quality of life scale

It evaluates the quality of life index of the users in the dimensions of emotional well-being, interpersonal relationships, material well-being, personal development, physical well-being, self-determination, social inclusion, and rights.^46^ The items of each dimension are rated using a four-option frequency scale. Standard scores (*M* = 10; standard deviation [SD] = 3) for each quality of life dimension, percentiles, and quality of life index. A quality of life index of 52 corresponds to the <1st percentile, while a quality of life index of 138 corresponds to the >99th percentile. The Cronbach’s alpha coefficient of validity is (0.91).

#### The Rey auditory verbal learning test

It measures verbal learning ability and short- and long-term retention. The version adapted for the Latino population was used.^47^ A target list of 15 words is read to the patient, who must try to remember it during five consecutive trials. Subsequently, a distracting list is presented, and the short-term memory of the target list is assessed. After 30 minutes, long-term memory of the words on the list is evaluated. The maximum value is 15 and the minimum value is 0. Means of references by age and schooling were controlled based on the reference population. Verbal learning and memory are assessed using the learning curve, total acquisition or total learning (I–V), and trials VI (working memory), VII (delayed recall), and VIII (recognition). The Cronbach’s alpha coefficient of validity is (0.80).

#### Digits-WAIS IV

It is used to measure immediate memory and working memory capacity.^48^ It involves repeating a series of numbers of increasing complexity, starting in sequential order, then in reverse order, and finally in increasing order. Wechsler Adult Intelligence scale (WAIS-IV) subtests were employed based on age. A score below 7 indicates limited cognitive function. The Cronbach’s alpha coefficient of validity is (0.94).

#### Coding—subtest WAIS-IV

The task involves completing squares with the appropriate symbols based on the digit in their upper part.^48^ It evaluates speed and visual-motor skills. WAIS-IV scales were used according to age. A score below 7 indicates limited cognitive function. The Cronbach’s alpha coefficient of validity is (0.94).

#### Test D2

It measures selective and sustained attention. The test contains 14 lines with 47 characters, totaling 658 items.^49^ These stimuli include the letters “d” or “p” accompanied by one or two small lines, individually or in pairs, located at the top or bottom of each letter. The subject’s task is to carefully review the content of each line from left to right, then mark every letter “d” that has the assigned designation or key, discriminating the relevant elements from the irrelevant ones. The direct scores obtained in overall test efficiency (*M*: 430.71; SD: 99.75); concentration index (*M*: 172.64; SD: 48.30); variation index (*M*: 14.57; SD: 6.13) were considered, according to the reference population. The Cronbach’s alpha coefficient of validity is (0.80–0.97).

#### Trail-making test, part A and part B

This test allows for the assessment of visual-motor speed and cognitive flexibility.^50^ Part A involves executing a task, connecting numbers in chronological order from 1 to 25 without releasing the line. Part B consists of executing a more complex task in which numbers and letters must be connected in an alternating increasing order (1 a, 2 b… 13 in sequential order) under time pressure, and the number of mistakes made is counted. A standardized version for the Latin population was used. The direct scores obtained in seconds were considered based on the means obtained in the reference population, controlling for age and schooling. The Cronbach’s alpha coefficient of validity is (0.70–0.90).

#### Stroop test

It is an instrument that assesses complex attention through the ability to inhibit verbal interference or faster automatic response, making it a good measure of selective attention.^51^ The level of interference was determined based on the scores obtained, with a typical score ranging from 20 to 80. The results were interpreted according to the means obtained in the reference population. The Cronbach’s alpha coefficient of validity is (0.89).

#### Phonological Verbal Fluency Test

It consists of a controlled and programmed verbal production test in which the subject must produce words that begin with a letter pre-established by the examiner for 1 minute (e.g., P-M-R).^52^ The scores were obtained directly and analyzed based on the means obtained in the reference population. The Cronbach’s alpha coefficient of validity is (0.82).

#### Semantic verbal fluency test

Assesses the ability to retrieve stored semantic information. It is related to the speed of organizing thoughts and the strategies used for the rapid search of words within a time frame of 60 seconds.^52^ The scores were obtained directly and analyzed based on the means obtained in the reference population. The Cronbach’s alpha coefficient of validity is (0.82).

### Data analysis

Data analysis was performed using the statistical software SPSS v.26.0. Initially, an analysis of basic descriptive data (central tendency [mean] and dispersion [standard deviation]) was conducted with the objective of obtaining a sociodemographic profile of the participants. Next, the variables of the neuropsychological tests (*z*) were standardized, and a frequency table (percentages) was created for the clinical variables. Subsequently, contingency tables were generated, and a regression analysis was conducted to estimate the relationships between variables using normality (Kolmogorov–Smirnov), parametric (Student *t*), and nonparametric (chi-square, Mann–Whitney) tests to assess differences between the pre- and post-treatment results. As a final analysis, the effect size of the differences found was measured through the Cohen statistic.

## Results

Figure 1 shows the CONSORT statement and provides the flowchart of the patient inclusion process. Table 3 shows the sociodemographic data of the participants.

**Table 3.** Comparison of Sociodemographic Characteristics of the Participants of the Eye Movement Desensitization and Reprocessing and Narrative Exposure Therapy Group

Variables	EMDR group (*N* = 51)	TEN group (*N* = 54)	p*
Age (M; SD)	35.2; 8.1	33.4; 8.3	0.644
Years of schooling (M; SD)	7.8; 3.1	9.1; 7.8	0.225
Violence exposure time (M; SD)	6.2; 4.7	6.6; 4.6	0.261
Physical violence (F; %)	23; 45.1	25; 46.3	0.990
Psychological violence (F; %)	22; 43.1	23; 42.6	
Sexual violence (F; %)	6; 11.8	6; 11.1	
Participants living without a partner (F; %)	11; 21.6	11; 20.4	0.997
Participants living with a partner (F; %)	40; 78.4	43; 79.6	

*There are no significant differences in the distribution of the EMDR and NET groups; they originate from equal distributions before being assigned to therapeutic treatments.

M, mean statistic; SD, standard deviation.

### Effectiveness of EMDR therapy compared with NET in reducing clinical symptomatology

Table 4 shows that the group of women treated with EMDR exhibited greater improvement in the Hospital Anxiety and Depression Scale (HADS), anxiety subscale, between pre- and post-treatment (*M* = 9.71; SD = 4.59), compared with the average values of the group treated with NET (*M* = 4.98; SD = 3.37), in the reduction of clinical anxiety (*p* = 0.001), with a moderate effect size (*d* = 0.62). Likewise, patients addressed with EMDR showed greater changes in reducing depression levels measured in the HADS, depression subscale (*M* = 6.1; SD = 3.5), compared with the average shown by the group of women treated with NET (*M* = 4.9 SD = 3.3); significant differences were evident between pre- and post-treatment (*p* = 0.001), with a moderate effect size (*d* = 0.51). Similarly, the average change values obtained between pre- and post-treatment in the EMDR group were more effective than NET in reducing post-traumatic stress, measured by the Post-Traumatic Stress Disorder Checklist for DSM-5 (*p* = 0.002), with a moderate effect size (*d* = 0.59). Finally, the group of women treated with EMDR showed more changes between pre- and post-treatment, on average (*M* = −23.3; SD = 13.9), compared with the average achieved by the NET group (*M* = −9.1; SD = 9.2), increasing the quality of life index, measured on the GENCAT Quality of Life scale (*p* = 0.001), and reporting moderate effect sizes (*d* > 0.52) (Table 4).

**Table 4.** Effectiveness of Eye Movement Desensitization and Reprocessing Therapy Compared with Narrative Exposure Therapy in Reducing Clinical Symptomatology

Clinical variables	Pre-treatment			Post-treatment			Differences			
	EMDR *N* = 51	NET *N* = 54	Student’s *t* *p*^a^	EMDR *N* = 51	NET *N* = 54	Student’s *t* *p*^b^	EMDR *N* = 51	NET *N* = 54	Student’s *t* *p*^c^	Cohen’s d^d^
	M; SD	M; SD		M; SD	M; SD		M; SD	M; SD		
HADS-A	11.2; 3.7	11.4; 3.9	0.702	1.5; 2.4	6.4; 3.2	0.001^**^	9.7; 4.5	4.9; 3.3	0.001^**^	**0.62**
HADS-D	8.8; 4.0	8.1; 3.8	0.485	1.9; 2.7	6.7; 3.2	0.001^**^	6.1; 3.5	1.4; 2.2	0.001^**^	**0.51**
PCL-5	49.8; 7.9	50.0; 7.8	0.939	6.0; 3.7	16.1; 6.9	0.002^*^	44.0; 9.9	34.1;11.4	0.002^*^	**0.59**
GENCAT	93.7; 15.9	94.0; 15.7	0.911	117.1; 9.8	103.2; 16.3	0.001^**^	−23.3; 13.9	−9.1; 9.2	0.001^**^	**0.52**

*p < 0.05; **p < 0.001.

^a^ Student’s *t-*test does not show significant differences *p* > 0.05 in the means obtained by the EMDR and NET groups at the beginning of the treatment.

^b^ Student’s *t-*test shows significant differences *p* < 0.05 in the means obtained by the EMDR and NET groups in the post-treatment evaluation.

^c^ Student’s *t-*test shows significant differences *p* < 0.05 in the means obtained by the EMDR and NET groups in the changes observed between pre- and post-treatment.

^d^ Cohen’s *d* shows the effect size of the treatment.

Student *t*: Student’s *t*-distribution plays a role in a number of widely used statistical analyses, including Student’s *t*-test for assessing the statistical significance of the difference between two sample means, the construction of confidence intervals for the difference between two population means, and in linear regression analysis. Cohen’s d: Cohen’s d is a standardized effect size for measuring the difference between two group means. *p* value: In general statistics and hypothesis testing, the *p* value is defined as the probability that a calculated statistical value is possible given a true null hypothesis.

HADS-A, Hospital Anxiety and Depression Scale (Anxiety); HADS-D, Hospital Anxiety and Depression Scale (Depression); PCL-5, Post-Traumatic Stress Disorder Checklist for DSM-5; GENCAT, Quality of Life scale.

### Effectiveness of EMDR compared with NET in enhancing cognitive performance among groups of women adhering to therapeutic treatment

In Table 5, the significant differences found between EMDR and NET treatments are presented, using the independent samples *t*-test. The group of women treated with EMDR showed greater changes in the indirect digits subtest, compared with the average values of the group treated with NET, in working memory performance (*p* = 0.001)*;* reporting a moderate effect size (*d* = 0.67). Likewise, EMDR showed greater changes in the averages of the Direct Digits test compared with NET, in tasks that assess working memory (*p* = 0.001); reporting a moderate effect size (*d* = 0.67).

**Table 5.** Effectiveness of Eye Movement Desensitization and Reprocessing Compared with NET in Enhancing Cognitive Performance among Groups of Women Adhering to Therapeutic Treatment

Variable	Test	Pre-treatment			Post-treatment				Differences		EMDR
		EMDR *N* = 51	NET *N* = 54	Student’s *t* *p*^a^	EMDR *N* = 51	NET *N* = 54	Student’s *t* *p*^b^	EMDR *N* = 51	NET *N* = 54	Student’s *t* *p*^c^	Cohen’s d effect size (*r*)^d^
		M; SD	M; SD		M; SD	M; SD		M; SD	M; SD		
Verbal memory	RAVLT	−1.2; d = 0.22	−1.16; 0.92	0.620	−0.63; 0.59	−0.48; 0.90	0.516	0.62; 0.65	0.63; 0.43	0.415	0.34
Working memory	Indirect digits	−1.4; 0.77	−1.6; 0.58	0.464	−0.31; 0.34	−1.53; 0.42	0.001^**^	1.16; 0.69	0.09; 0.36	0.001^**^	**0.67**
	Direct Digits	−1.2; 0.64	−1.3; 0.45	0.375	−0.35; 0.57	−1.33; 0.46	0.001^**^	0.90; 0.63	−0.01; 0.42	0.001^**^	**0.57**
Attention	D2	−1.2; 1.07	−1.3; 1.22	0.941	−1.12; 1.07	−1.22; 1.23	0.736	0.16; 0.23	0.11; 0.10	0.150	0.03
Processing speed	Coding	−1.26; 0.91	−1.34; 1.01	0.592	−0.40; 0.55	−0.99; 0.76	0.001^**^	0.16; 0.23	0.11; 0.10	0.518	0.49
Executive functions	TMT-A	−1.3; 3.31	−2.5; 2.35	0.020^*^	1.54; 3.23	1.98; 2.20	0.027^*^	0.86; 0.68	0.35; 0.54	0.001^**^	0.22
	TMT-B	−1.5; 2.04	−1.6; 1.57	0.274	1.1; 2.03	1.44; 1.54	0.104	−0.37; 0.21	−0.24; 0.20	0.001^**^	0.24
	Stroop	−1.55; 0.76	−1.51; 0.93	0.939	−0.12; 0.71	−1.36; 0.81	0.001^**^	1.42; 0.79	0.15; 0.55	0.001^**^	**0.69**
	SVFT	−0.95; 0.74	−0.72; 0.83	0.327	3.1; 1.65	1.22; 1.27	0.001^**^	4.12; 1.67	1.96; 1.40	0.001^**^	**0.64**
	PVFT	−1.31; 1.22	−0.94; 1.07	0.054	0.75; 1.27	−0.12; 0.84	0.001^**^	2.07; 1.12	0.82; 0.94	0.001^**^	0.34

*p < 0.05; **p < 0.01.

^a^ Student’s *t-*test does not show significant differences *p* > 0.05 in the means obtained by the EMDR and NET groups at the beginning of the treatment.

^b^ Student’s *t-*test shows significant differences *p* < 0.05 in the means obtained by the EMDR and NET groups in the post-treatment evaluation.

^c^ Student’s *t-*test shows significant differences *p* < 0.05 in the means obtained by the EMDR and NET groups in the changes observed between pre- and post-treatment.

^d^ Effect size of EMDR therapy comparing changes between pre- and post-tests.

Coding, WAIS-IV coding subtest; D2, test D2; indirect digits, WAIS-IV indirect digits subtest; WAIS-IV direct Digits subtest; PVFT, phonetic verbal fluency test; RAVLT, the Rey auditory verbal learning test; Stroop, Stroop test; SVFT, semantic verbal fluency test; TMT, trail-making test.

Regarding tests that assess executive functioning, significant differences were observed. In the trail-making test (TMT)-A test, women treated with EMDR showed greater changes compared with those who received NET treatment in sustained attention tasks (*p* = 0.001), reporting a small effect size (*d* = 0.22). EMDR also proved to be more effective than NET, improving cognitive flexibility in treated women, as evidenced by pre–post changes in the TMT-B test (*p* = 0.004), reporting a small effect size (*d* = 0.22). Additionally, EMDR showed greater efficacy compared with NET in increasing inhibition capacity and resistance to interference, as demonstrated in the Stroop test (*p* = 0.001), reporting a moderate effect size (*d* = 0.69). Finally, EMDR demonstrated greater effectiveness in improving semantic verbal fluency skills compared with the performance achieved in the NET-treated group, as confirmed by the semantic verbal fluency test (SVFT) test (*p* = 0.001), reporting a moderate effect size (*d* = 0.64).

### Differences in the response to EMDR treatment among the studied sociodemographic factor groups

Given that in the previous sections it was demonstrated that EMDR is more effective than NET, we wanted to verify if the effectiveness of EMDR showed dependence on the studied sociodemographic factors. Under this assumption, Table 6 shows that women with 8 years or less of education exhibited greater changes in the pre- and post-test, within tests measuring processing speed (coding test) and verbal fluency (SVFT, phonetic verbal fluency test). Also, the group of women exposed to physical violence showed greater changes between pre- and post-comparisons in attentional performance (D2 test) and executive performance (TMT-B). No significant differences were observed in the other sociodemographic factors (*p* < 0.05).

**Table 6.** Differences in the Response to Eye Movement Desensitization and Reprocessing Treatment among the Studied Sociodemographic Factor Groups

Variables	Test	Age		*U* de Mann–Whitney *p*^a^	Years of schooling		*U* de Mann–Whitney *p*^b^	Marital status		*U* de Mann–Whitney *p*^c^	Physical violence *n* = 23)	Psychological violence *n* = 22	Sexual violence *n* = 6)	Kruskal–Wallis *p*^d^
		18–30 years *n* = 12	31–50 years *n* = 39		8 years of schooling or less *n* = 31	9 years of schooling or more *n* = 19		Living without a partner *n* = 11	Living with a partner *n* = 40					
		M; DS	M; DS		M;DS	M;DS		M; DS	M; DS		M;DS	M; DS	M; DS	
Verbal memory	RAVLT	0.58; 0.71	0.64; 0.64	0.743	0.68; 0.66	0.51; 0.65	0.393	0.40; 0.70	0.68; 0.63	0.300	0.74; 0.64	0.63; 0.64	0.01; 0.61	0.176
Working memory	Indirect Digits	1.08; 0.85	1.1; 0.64	0.820	1.31; 0.70	0.91; 0.63	0.231	1.42; 0.74	1.09; 0.67	0.325	1.14; 0.79	1.24; 0.62	0.94; 0.53	0.301
	Direct Digits	0.97; 0.65	0.88; 0.63	0.964	1.02; 0.69	0.68; 0.48	0.060	1.33; 0.55	0.78; 0.61	0.**015**	0.72; 0.64	1.07; 0.58	0.94; 0.74	0.301
Attention	D2	0.08; 0.43	0.19; 0.11	0.505	0.18; 0.13	0.13; 0.35	0.992	0.07; 0.39	0.18; 0.16	0.663	0.23; 0.18	0.13; 0.28	0.02; 0.004	0.002^**^
Processing speed	Coding	0.94; 0.56	0.82; 0.71	0.676	1.05; 0.60	0.45; 0.56	0.001^**^	0.72; 0.44	0.89; 0.73	0.429	0.85; 0.80	0.97; 0.58	0.44; 0.34	0.136
	TMT-A	−0.61; 0.61	−0.32; 0.30	0.289	−0.41; 0.32	−0.35; 0.54	0.140	−0.53; 0.55	−0.3; 0.36	0.462	−0.33; 0.41	−0.49; 0.45	−0.02; 0.14	0.235
Executive functions	TMT-B	−0.30; 0.23	−0.38; 0.20	0.105	−0.36; 0.21	−0.37; 0.21	0.749	−0.25; 0.17	−0.39; 0.21	0.057	−0.45; 0.23	−0.33; 0.16	−0.15; 0.05	0.001^**^
	Stroop	1.57; 0.69	1.37; 0.82	0.601	1.44; 0.84	1.37; 0.75	0.719	1.29; 0.71	1.46; 0.82	0.528	1.23; 0.88	1.66; 0.72	1.26; 0.45	0.212
	SVFT	3.52; 1.24	4.29; 1.75	0.239	4.82; 1.74	2.96; 0.65	0.001^**^	3.8; 1.83	4.2; 1.63	0.347	4.01; 2.02	4.20; 1.31	4.17; 1.58	0.577
	PVFT	1.83; 1.06	2.14; 1.15	0.339	2.56; 1.09	1.24; 0.62	0.001^**^	1.9; 1.12	2.1; 1.14	0.655	2.18; 1.08	2.22; 1.13	1.10; 0.91	0.108
Anxiety	HADS-A	9.92; 3.5	9.64; 4.9	0.867	9.97; 3.41	9.32; 6.23	0.944	7.4; 6.23	10.3; 3.9	0.138	9.70; 4.05	9.59; 5.6	10.17; 2.40	0.936
Depression	HADS-D	6.25; 3.10	6.1; 3.7	0.568	6.68; 3.08	5.32; 4.23	0.319	6.0; 2.91	6.20; 3.85	0.972	6.39; 3.20	5.64; 3.63	7.17; 4.75	0.841
Post-traumatic stress	PCL-5	42.92; 10.97	44.3; 9.70	0.911	43.5; 9.60	44.1; 10.53	0.624	47.3; 5.8	43.0; 10.64	0.184	46.52; 10.48	43.23; 9.33	37.17; 6.85	0.095
Quality of life	GENCAT	29.5; 12.75	21.51; 13.97	0.117	24.4; 14.9	21.9; 12.9	0.477	21.3; 7.7	23.9; 15.30	0.477	20.39; 15:43	27.86; 12.93	18.50; 7.03	0.145

*p < 0.05; **p < 0.01.

^a^ The Mann–Whitney *U* test shows that there are no significant differences when comparing the means obtained in the age groups.

^b^ The Mann–Whitney *U* test shows the significant differences observed when comparing the groups based on years of schooling.

^c^ The Mann–Whitney *U* test shows the significant differences observed when comparing the groups by marital status.

^d^ The Mann–Whitney *U* test shows the significant differences observed when comparing the means of the groups by type of violence, in the response to EMDR treatment.

Kruskal–Wallis *p* value, nonparametric test that measures differences in means reported in three categorical groups; *U* de Mann–Whitney, nonparametric test estimating the difference in means between two groups.

## Discussion

The literature has shown the existence of various therapies based on narrative approaches such as NET and other therapies under the cognitive-behavioral paradigm, which have been suggested for the intervention of women victims of violence, some with more comprehensive approaches and others still with limitations in reports related to their effectiveness.^19,20,53^

The results found in our study reported that EMDR was more effective compared with NET in reducing clinical symptoms, increasing cognitive performance, and improving the quality of life index in women exposed to physical, psychological, or sexual violence. Previous literature has shown studies of women victims of violence from diverse sociocultural contexts who demonstrated improvement after undergoing EMDR-based treatment.^29–31^ Regarding NET, no studies specifically addressed battered women; however, it is a therapy applied in the Ecuadorian context, as it has shown effectiveness in reducing post-traumatic symptoms.^22,54^

Analyzing the results of the research and becoming aware of the adverse effects that violence generates in women, affecting their mental health,^1–3^ it could be suggested that, in addition to NET therapy, which is part of Ecuador’s public health services, EMDR therapy be included. This is because it showed greater changes in the therapeutic response of Ecuadorian women who were part of the study, overcoming sociodemographic factors associated with the issue of violence. Additionally, it could potentially result in lower economic costs.^55^

Moreover, there are few psychological therapies within the Ecuadorian context that are indicated for the integrated approach to the mental health care of victims of violence, given that psychological sequelae are often multiple.^3–19^ Similarly, NET appears more limited based on the literature supporting its effectiveness, relying on its mechanism of action from neurobiological and neurophysiological perspectives, using techniques such as brain mapping and electroencephalograms to provide greater clarity and effectiveness regarding its therapeutic scope. This is in contrast to the EMDR model, which has many years of supporting its mechanism of action in the brain through various approaches, including neuropsychological, biological, physiological, and somatic perspectives.^56,57^

The findings uncovered open up broad avenues for future research concerning therapies that contribute to the comprehensive rehabilitation of victims of violence. Given that the 2030 Agenda, under Sustainable Development Goal 3 focusing on health and well-being, calls on the Ecuadorian Ministry of Health to ensure the right to health for women who have experienced violence and to eradicate all forms of violence. In this way, serious consideration should be given to the recommendations made by the WHO,^27^ who have emphasized the utility of EMDR in the treatment of individuals who are victims of violence.

### Limitations of the research

This research had limitations due to the absence of a control group, and the study participants were from a rural cultural context. Despite the number of participants falling within the standards proposed by the scientific community for this type of clinical study, it may be limiting for result generalization. Therefore, future research is recommended to expand the sample size and include women from different sociocultural contexts for a more comprehensive comparison between these groups.

Additionally, NET therapy exhibits greater limitations both in previous studies supporting its efficacy in abused women and in its application protocol, which is simpler than EMDR. Its inclusion in this study for comparison with EMDR is justified, given that narrative approaches are commonly used in most Ecuadorian healthcare services.

## Conclusions

EMDR proved to be more effective compared with NET in reducing clinical symptoms associated with anxiety, depression, and post-traumatic stress.

The group of abused women treated with EMDR experienced a greater improvement in the quality of life index compared with the group of abused women treated with NET.

The group of women treated with EMDR showed better performance in working memory and executive functioning compared with the performance of the group treated with NET.

Most sociodemographic factors studied, such as age, years of education, marital status, and type of violence, did not affect the therapeutic response in patients undergoing EMDR therapy.

## Declarations

Yakenine Alexandra Meneses declares that part of this research comes from the doctoral thesis supported.

## Ethics Approval and Consent to Participate

The research received an approval from the Ethical Committee and Human Research of UTE University of Medical Sciences https://www.ute.edu.ec/comite-de-etica-de-investigacion-en-seres-humanos-ute-ceish/#1507826021858-0b8f2ecb-bb21 with a protocol identifier code of 040-CEISH-jcm. Informed consent has been obtained from the participants before the beginning of data collection. The authors confirm that all methods were performed in accordance with the Declaration of Helsinki—ethical principles for medical research involving human subjects.

## Abbreviations Used

DSM-V: Diagnostic and Statistical Manual of Mental Disorders
EMDR: Eye Movement Desensibilization and Reprocessing
HADS: Hospital Anxiety and Depression Scale
ICD-11: International Classification of Diseases-eleventh Edition
IQ: Intel.ligence Quotient
M: Mean
NART: National Adult Reading test
NET: Narrative Exposure Therapy
PTSD: Post Traumatic Stress Disorder
RAVLT: Rey Auditory Verbal Learning Test
SD: Standard Desviation
SPSS: Social Sciences Statistical Package
SUB: Subjective Unints of Disturbance
SVFT: Semantic Verbal Fluency test
TMT: Trail Making Test
TMT-A: Trail Making test part A
TMT-B: Trail Making test part B
UTE: Universidad Tecnológica Equinoccial
VoC: Cognition Validity Scale
WAIS: Wechsler Adult Intelligent Test
WAIS IV: Wechsler Adult Intelligent Test Fourh Edition
WHO: World Health Organization
