# Supplementary material for: Eye Movement Desensitization and Reprocessing: Efficacy in Improving Clinical, Neuropsychological, and Quality of Life in Women Victims of Violence
Source: Womens Health Rep (New Rochelle). 2024 Dec 6;5(1):984–96. doi: 10.1089/whr.2023.0110 (PMC11693959; doi:10.1089/whr.2023.0110)
Supplement: Supplementary Figure S1 [file whr.2023.0110_supp_figs1.doc]

**
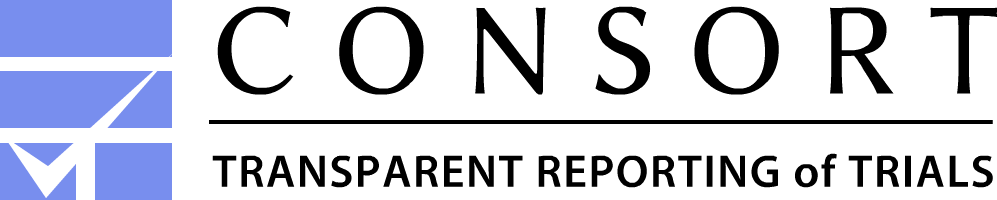
**

**Figure 1. CONSORT Flow Diagram**

**Allocation**

**Analysis**

**Follow-Up**

**Enrollment**

Assessed for eligibility (n=170)

Excluded (n=50)

♦  Not meeting inclusion criteria (n=40)

♦  Declined to participate (n=10)

♦  Other reasons (n=0)

Analysed (n= 51)
♦ Excluded from analysis (They did not complete the treatment) (n=9)

Lost to follow-up (associated with the Covid-19) (n=9)

Allocated to intervention EMDR (n=60)

♦ Received allocated intervention (n=60 )

Lost to follow-up (associated with the Covid-19) (n=6)

Allocated to intervention (n= 60)

♦ Received allocated intervention (n=60)

Analysed (n= 54)
♦ Excluded from analysis (They did not complete the treatment) (n=6)

Randomized (n=120)
